# Anxiety Assessment in Polish Students during the Russian–Ukrainian War

**DOI:** 10.3390/ijerph192013284

**Published:** 2022-10-14

**Authors:** Edyta Skwirczyńska, Mateusz Kozłowski, Katarzyna Nowak, Oskar Wróblewski, Agnieszka Sompolska-Rzechuła, Sebastian Kwiatkowski, Aneta Cymbaluk-Płoska

**Affiliations:** 1 Department of History of Medicine and Medical Ethics, Pomeranian Medical University in Szczecin, 70-204 Szczecin, Poland; 2 Department of Gynecological Surgery and Gynecological Oncology of Adults and Adolescents, Pomeranian Medical University in Szczecin, Al. Powstańców Wielkopolskich 72, 70-111 Szczecin, Poland; 3 Doctoral School, Pomeranian Medical University in Szczecin, 70-204 Szczecin, Poland; 4 Department of Applied Mathematics in Economics, Faculty of Economics, West Pomerania University of Technology Szczecin, Janickiego 31, 71-270 Szczecin, Poland; 5 Department of Obstetrics and Gynecology, Pomeranian Medical University in Szczecin, Al. Powstańców Wielkopolskich 72, 70-111 Szczecin, Poland

**Keywords:** anxiety, STAI, students, war, Russian-Ukrainian War, logit

## Abstract

Anxiety is described as a feeling of fear that appears in stressful or threatening situations. A terrorist attack is one such situation. The aim of this study was to assess anxiety levels among students using the STAI questionnaire. The study group consisted of 510 participants. Statistical analysis was performed using Statistica software. Anxiety levels, correlations between STAI scores, and individual variables were assessed. Logit models were performed for the study variables. Higher levels of anxiety were found in females compared to males (*p* = 0.0000). The highest level of anxiety overall was found in year 1 students, the lowest in year 5 students (*p* = 0.0005). The highest level of anxiety overall was found in pharmacy students, the lowest in midwifery students. We concluded that there was a relatively strong relationship between anxiety and gender. Gender and fear of an armed attack on Poland had a significant impact on anxiety.

## 1. Introduction

Anxiety is a disconcerting subjective experience defined as the expectation of future danger [1,2]. It is also described as a feeling of fear that appears in stressful or threatening situations. It is considered that if the anxiety is persistent or overwhelming, one can already speak of anxiety disorder [3]. While anxiety can be a normal emotion, it can also be a symptom of many illnesses, including depression or posttraumatic stress disorder (PTSD) [4]. Anxiety can also be distinguished as a temporary, transient emotional state (anxiety as a state) and anxiety understood as a persistent, permanent personality trait, expressed in the readiness to react with anxiety in certain situations (anxiety as a trait) [5].To measure anxiety levels research studies use, among others, the State-Trait Anxiety Inventory (STAI), which assesses anxiety precisely as a trait and as a state, or Generalized Anxiety Disorder (GAD-7) questionnaire, which assesses anxiety over the past two weeks [6]. In addition to assessing anxiety itself, researchers, in their work on human behavior, also evaluate coping strategies for example by using scales such as the Coping Orientation to Problems Experienced (COPE) [7,8].

Both children and adolescents are a group at increased risk of developing symptoms and anxiety disorders. The same applies to women, who are twice as likely as men to develop anxiety disorders [9]. Another group at risk of increased levels of anxiety are also students, who are affected by anxiety both under normal conditions and in crisis situations, such as pandemics or armed conflicts [10,11,12,13,14].

As some of the many causes of anxiety, terrorist attacks or the ongoing COVID-19 pandemic may be considered [15,16]. Anxiety can also be induced by a state of war. [8] It can then affect residents of the country at war, soldiers fighting on the front lines, and also people indirectly impacted by the events of armed conflicts, such as friends and relatives of those affected by the war or residents of neighboring countries [17]. It is estimated that worldwide, in 2022 there are more than 100 million displaced people [18]. Some of them are war refugees. In February 2022, the Russian–Ukrainian war began, during which millions of Ukrainian citizens became refugees who fled outside their country’s borders in fear of war. Most refugees from Ukraine found safe haven in countries bordering Ukraine, including Poland, Romania, and Moldova [19]. Data released in early October showed that more than 6.5 million refugees had crossed the Polish–Ukrainian border since the start of the war. Most refugees remain in their host countries for years after leaving their country [20]. The effects of the war have and will continue to have a negative impact on the mental health of Ukrainian citizens. This includes both soldiers and civilians [21]. However, anxiety and fear also accompany the residents of other countries, including Poland. Such a large influx of people from war-torn Ukraine, in addition to the information reported in the media, has contributed to growing anxiety among Polish citizens. The aim of this study was to assess anxiety levels among students at universities in Szczecin using the State-Trait Anxiety Inventory (STAI). The following hypotheses were posed.

1. Anxiety levels correlate with gender.
2. If the person is a woman or a senior student, then the odds of an increase in anxiety levels decrease.
3. If a person fears an armed attack by Russia on Poland, then the odds of increasing anxiety increase.

## 2. Materials and Methods

### 2.1. Study Design, Participants, and Selection Criteria

The survey was conducted between 1 March 2022 and 30 March 2022. The participants in the survey were students of the Pomeranian Medical University in Szczecin (Poland). The survey was conducted online using the CAWI technique. Participation in the study and completion of the questionnaire were voluntary and anonymous.

The study group consisted of students of the faculties of medicine and dentistry, pharmacy, midwifery, nursing, dietetics, psychology, and physiotherapy at the Pomeranian Medical University in Szczecin. Participants completed an online questionnaire on sociodemographic data, perceived anxiety understood as a temporary and situationally conditioned state of the individual, and anxiety understood as a relatively permanent personality trait. The study group consisted of 510 participants: 402 medical students, 66 dental students, 13 pharmacy students, 14 midwifery students, 7 nursing students, 2 dietetics students, 3 psychology students, and 3 physiotherapy students. Seven students did not answer what faculty they were in, and these questionnaires were not included in the survey.

The inclusion criteria for the study were: age 18 and over, being a student of the Pomeranian Medical University in Szczecin, and willingness to participate in the study. The exclusion criteria for the study were: under 18 years of age and incomplete filling of the questionnaire.

### 2.2. Instruments

The questionnaire consisted of three parts. The first part consisted of sociodemographic data such as age, gender, faculty, year of study, nationality and religion. The second part consisted of the following questions:

- Are you afraid of an armed attack by Russia on Poland?
- Are you stocking up? If yes—then what?
- Do you have cash savings?
- Did you withdraw your savings money?
- If you had savings, would you withdraw them from the bank?
- Which currency do you trust the most?
- In the event of the armed conflict in Poland, would you take an active part in it?
- Would you leave Poland in the event of armed conflict? If yes—to which country?
- Are you taking an active part in helping Ukraine?
- In your opinion, is the aid to Ukraine sufficient?

For the questions “Are you stocking up? If yes—then what?”, “Which currency do you trust the most?”, and “Would you leave Poland in the event of armed conflict? If yes—to which country?”, more than one answer was possible. The third part consisted of the STAI (State-Trait Anxiety Inventory) questionnaire. The questionnaire consists of 40 statements and is divided into two subscales of 20 questions each. The first 20 statements relate to anxiety as a state (s-STAI), which assesses a transient emotional state; the second 20 statements relate to the anxiety as a trait subscale (t-STAI), which, in general, considers a relatively stable propensity towards anxiety. The entire questionnaire uses a 4-point Likert scale (0—almost never/not at all; 1—sometimes/somewhat; 2—often/moderately so; 3—almost always/very much so). The range of possible scores for the STAI varies from a minimum score of 20 to a maximum score of 160. We used the STAI in English and had it translated into Polish by a language expert.

The reliability of the scale was assessed using Cronbach’s alpha coefficient and split-half reliability, which takes values from 0 to 1. The scale is reliable if the reliability coefficients are larger than 0.6 and smaller than 1.

### 2.3. Statistical Methods

Statistical analysis was performed using Statistica software. Three types of statistical methods were used in analyzing the collected research material and achieving the aim stated. In the first step, statistical description methods were used to characterize the data, determining the values of such parameters as the number of valid cases, arithmetic mean, median, minimum, maximum and asymmetry coefficient. Because the distribution of the age variable was not a normal distribution and the other variables were expressed on an ordinal scale at most, we used nonparametric significance tests: the Mann–Whitney U test (for two populations), Kruskal–Wallis ANOVA (for more than two populations), and the post hoc test of Dunn [22,23,24]. A logistic regression model, in which the dependent variable was dichotomous and the independent variables could be both qualitative and quantitative, was used to isolate the set of variables that significantly affect the probability of increasing anxiety [25]. An assessment of the degree of fit of the logistic regression model to empirical data was carried out using the measure called $R_{count}^{2}\;$ and receiver-operating characteristic (ROC) curves [26].

### 2.4. Ethics

All participants completed the questionnaire voluntarily and anonymously. The study was conducted in accordance with the Declaration of Helsinki and approved by the Ethics Committee of the Pomeranian Medical University in Szczecin (protocol code KB-0012/97/2020).

## 3. Results

### 3.1. Group Characteristics

The results obtained from the research using the STAI questionnaire were verified for scale reliability using the Cronbach’s alpha coefficient and split-half reliability. The values of the coefficients for the entire scale were 0.647 and 0.784, respectively. For the STAI, t-STAI, and s-STAI variables, the Cronbach’s alpha coefficient was 0.939, while in the case of the war-related variables, it was slightly lower and amounted to 0.681.

The split-half reliability coefficient was 0.892 and the halves strongly correlated with each other—the correlation coefficient was 0.805. In addition, the values of Cronbach’s alpha coefficients in individual e-heads assumed satisfactory values—0.605 and 0.633.

In the survey conducted, the age distribution of respondents was characterized by strong right-sided asymmetry. The median age of participants was 21 years. In the study, 65% of participants were female (*n* = 330), while 35% were male (*n* = 180). The largest group was made up of medical (*n* = 402, 78%) and dentistry (*n* = 66, 13%) students. Just over half (53%) of the participants in the survey were first-year students and 22% were third-year students. Polish nationality accounted for 98% of the survey participants. Taking religion into account, 61% of participants were Christian and 16% were atheist. Most survey participants (52%) responded that they feared an armed attack by Russia on Poland. One in four surveyed were making stocks such as food, fuel, water, personal protective equipment, medicines, or power banks.

The vast majority of respondents (74%) had cash savings and one in five had withdrawn cash savings. Survey participants responded that they trusted the following currencies the most: euro (60%), US dollar (17%), and Polish zloty (15%). In case of an armed conflict in Poland, 30% of respondents would have taken an active part in it, whereas 61% of respondents would have left Poland. They would have emigrated to Germany, the UK, US, or Switzerland.

Almost 60% of respondents took an active part in helping Ukraine and 49% believed that there was not sufficient aid to Ukraine. Table 1 shows the percentage of women and men by responses relating to the war situation.

Based on the information in Table 1, it can be concluded that the percentages of women and men differed significantly on the following issues: having cash savings, not withdrawing cash savings, actively participating in providing aid to Ukraine, and stating that Ukraine’s aid was insufficient.

### 3.2. Comparison of STAI Scores in the Study Subgroups

Significant differences were found in the distributions of STAI total (*p* = 0.0000), s-STAI I (*p* = 0.0000) and t-STAI (*p* = 0.0000) scores for females and males. The distributions of STAI total scores by year of study differed significantly (*p* = 0.0006). There were significant differences in the distributions between STAI total points for year 1 and year 5 students (*p* = 0.0004) and year 3 and year 5 students (*p* = 0.0005). It was found that the distributions of s-STAI scores by year of study were not equal (*p* = 0.0001). There were significant differences in the distributions between the number of points for people in years 1 and 5 (*p* = 0.0001) and 3 and 5 (*p* = 0.0000). The distributions of t-STAI scores by year of study showed no significant differences (*p* = 0.7035). The distributions of STAI total scores by faculty were not equal (*p* = 0.0179). The distributions of s-STAI scores for students by faculty differed significantly (*p* = 0.0206). There were differences in the distributions of scores for dental and medical students (*p* = 0.0148). In contrast, the distributions of t-STAI scores for students in each faculty showed no significant differences (*p* = 0.1885).

### 3.3. Correlations between STAI Scores and Individual Variables

The analysis conducted showed a relatively strong, significant correlation between s-STAI and t-STAI scores among all study participants (*r_s_* = 0.676), among women (*r_s_* = 0.619) and among men (*r_s_* = 0.698). This was a correlation in the positive direction, meaning that the higher the level of anxiety as a state, the higher the level of anxiety as a trait in all study populations. A significant weak correlation was found between total STAI and gender (*χ*^2^ = 34.77, *p* = 0.0000), s-STAI and gender (*χ*^2^ = 21.97, *p* = 0.0000), and t-STAI and gender (*χ*^2^ = 28.57, *p* = 0.0000). Correlations between STAI scores and faculty were nonsignificant and very weak (*χ*^2^ = 7.27, *p* = 0.4009). There were also significant weak correlations between STAI total and year of study (*χ*^2^ = 20.63, *p* = 0.0009) and t-STAI and year of study (*χ*^2^ = 36.67, *p* = 0.0000). Correlation between s-STAI and year of study (*χ*^2^ = 2.98, *p* = 0.7035) was nonsignificant. Correlations between STAI scores and religion were nonsignificant and very weak.

### 3.4. Parameter Evaluations for Logit Models

In the next step of the study, it was determined which variables significantly affected the probability of higher war-related anxiety. For this purpose, logit models were developed with the following set of independent variables:

- age (“1” for those over 21 and “0” for those under 21),
- gender (“K” for women, “M” for men),
- faculty of study (“medicine and dentistry” and “other”),
- year of study (“0” for students in years 1–3 and “1” for students in years 4–6,
- fear of an armed attack by Russia on Poland (“1”—Yes and “0”—No),
- nationality (“PL”—Poland and “Other”),
- religion (“Ch”—Christianity and “Other”),
- stockpiling (“1”—Yes, “0”—No),
- having cash savings (“1”—Yes and “0”—No),
- withdrawal of savings (“1”—Yes and “0”—No),
- withdrawal from a bank (“1”—Yes and “0”—No),
- active participation in Poland’s armed conflict (“1”—Yes and “0”—No),
- departure from Poland in case of armed conflict in Poland (“1”—Yes and “0”—No).

Active participation in helping Ukraine (“1”—Yes and “0”—No).

As dependent variables, the following were accepted:

- STAI total (“1” for scores higher than 94 and “0”—for lower than 94),
- s-STAI (“1” for number of points higher than 46 and “0”—for less than 46),
- t-STAI (“1” for number of points higher than 46 and “0”—for less than 46).

In order to find the best combination of variables significantly affecting the probability of higher war-related anxiety, a formal selection of variables was made, for each logit model, using backward regression.

#### 3.4.1. Logit Model for the Dependent Variable STAI Total

The logit model parameter estimates for the dependent variable STAI total including variables obtained using backward regression are shown in Table 2.

In the model, the following had a positive, statistically significant impact on the dependent variable STAI: gender and fear of an armed attack on Poland—if a person was female or feared an armed attack on Poland then the odds of increasing STAI total increased. Negative statistically significant effects on the dependent variable STAI had: year of study, religion and having savings—if a person was a student in at least the fourth year or professed Christianity or had savings, then the odds of increasing STAI total decreased.

By interpreting the odds ratios at the *i*-th variable (assuming that the other variables included in the model remain unchanged), the following information was obtained:

- if the person was female, then the chance of increasing STAI increased by 154%,
- if a person was a student of at least the fourth year, then the chance of increased STAI decreased by 54.50%,
- if a person feared an armed attack by Russia on Poland, then the chance of increased STAI increased almost fourfold,
- if a person professed Christianity, the chance of increased STAI decreased by 41.02%,
- if a person had savings, then the chance of increased STAI decreased by 48.32%.

The validity of the estimated model was assessed by counting the accuracy of the classification of people (Table 3).

The classification accuracy was assessed using the $R_{count}^{2}$ coefficient and the ROC curve. The $R_{count}^{2}$ coefficient took a value of 69.04% and was greater than 50%, so it could be concluded that classification based on the model was better than random.

**Figure 1:**
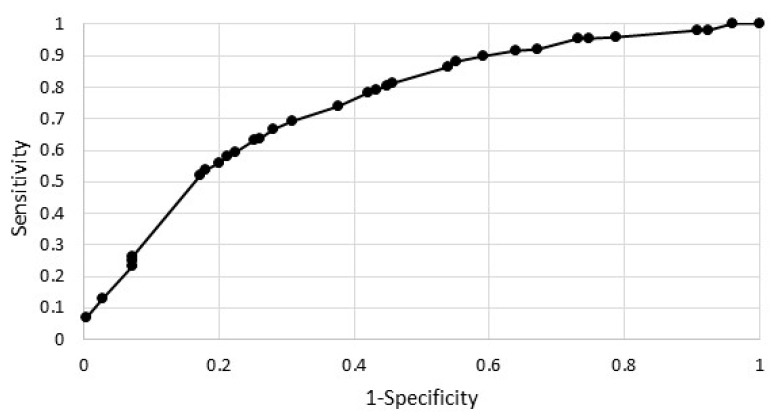
ROC curve.

Figure 1 shows the ROC curve for the estimated model. The area under the ROC curve was equal to 0.75 and significantly greater than 0.5 (for *p* = 0.0000).

The results of the Hosmer–Lemeshow test indicated that there were no significant differences between the empirical and theoretical abundances derived from the estimated logistic regression models (*χ*^2^ = 0.7718, *p* = 0.9977).

#### 3.4.2. Logit Model for the Dependent Variable s-STAI

The logit model parameter estimates for the dependent variable s-STAI, including variables obtained using backward regression, are shown in Table 4.

In the model, a positive, statistically significant impact on the dependent variable s-STAI had gender and fear of an armed attack on Poland—if a person was female or feared an armed attack on Poland, then the odds of increasing s-STAI increased. A negative, statistically significant impact on the dependent variable s-STAI had year of study—if a person was a student in at least the fourth year, then the odds of increasing s-STAI decreased.

Interpreting the odds quotients at the *i*-th variable (assuming that the other variables included in the model remained unchanged) yielded the following information:

- if the person was female, then the chance of increasing s-STAI increased by 112%,
- if the person was a student in at least the fourth year, then the chance of increasing s-STAI decreased by 71.02%,
- if a person feared an armed attack by Russia on Poland, then the chance of increasing s-STAI increased by more than almost three times.

The validity of the estimated model was assessed by counting the accuracy of the classification of individuals (Table 5).

The classification accuracy was assessed using the $R_{count}^{2}$ coefficient and the ROC curve. The $R_{count}^{2}$ coefficient was 72.27% and was greater than 50%, so it could be concluded that classification based on the model was better than random.

**Figure 2:**
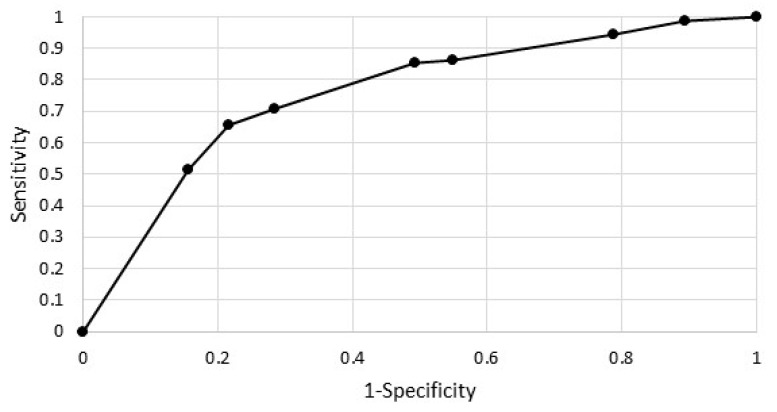
ROC curve.

Figure 2 shows the ROC curve for the estimated model. The area under the ROC curve was 0.75, significantly greater than 0.5 (for *p* = 0.0000).

The results of the Hosmer–Lemeshow test indicated that there were no significant differences between the empirical and theoretical counts from the estimated logistic regression models (*χ*^2^ = 7.5258, *p* = 0.1843).

#### 3.4.3. Logit Model for the Dependent Variable t-STAI

Logit model parameter estimates for the dependent variable t-STAI, including variables obtained using backward regression, are shown in Table 6.

In the model, a positive, statistically significant effect on the dependent variable t-STAI was found for gender and fear of an armed attack on Poland—if a person was female, studying in at least the fourth year or feared an armed attack on Poland, then the odds of increasing STAI increased. The other two variables had a negative, statistically significant effect on the dependent variable t-STAI—if a person had money in the bank and withdrew it or if he/she declared taking an active part in an armed conflict in Poland, then the odds of increasing t-STAI decreased.

By interpreting the odds ratios at the *i*-th variable (assuming that the other variables included in the model remained unchanged), the following information was obtained:

- if the person was female, then the odds of increasing t-STAI increased by 98.49%,
- if a person feared an armed attack by Russia on Poland, then the odds of increasing t-STAI increased by 164%,
- if a person professed Christianity, the chance of increased STAI decreased by 23%,
- if a person withdrew money from a bank, the odds of increased t-STAI increased by 115%,
- if a person declared taking an active part in an armed conflict, then the chance of increasing t-STAI decreased by 37.62%.

The validity of the estimated model was assessed by counting the accuracy of the classification of individuals (Table 7).

The classification accuracy was assessed using the $R_{count}^{2}$ coefficient and the ROC curve. The $R_{count}^{2}$ coefficient was 67.70% and was greater than 50%, so it could be concluded that classification based on the model was better than random.

**Figure 3:**
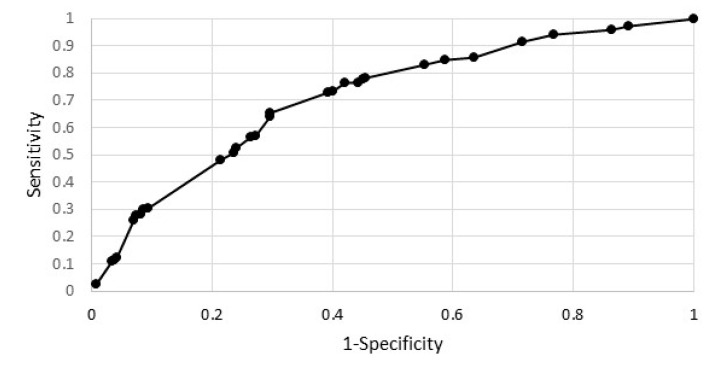
ROC curve.

Figure 3 shows the ROC curve for the estimated model. The area under the ROC curve was 0.71 and is significantly greater than 0.5 (for *p* = 0.0000).

The results of the Hosmer–Lemeshow test indicated that there were no significant differences between the empirical and theoretical counts from the estimated logistic regression models (*χ*^2^ = 9.2589, *p* = 0.2346).

## 4. Discussion

When preparing for their professional careers, university students have to confront psychosocial changes in addition to coping with the social and academic demands [27]. In addition to challenges from universities and peers in the form of exams, pressure to achieve the best possible results, and sometimes lack of family support, young people also struggle with anxiety caused by other circumstances. Our study consisted of assessing the level of anxiety among Polish students during the war in Ukraine. To the best of our knowledge, this is one of the few studies assessing anxiety among students during the Russian–Ukrainian War.

Women are more likely than men to develop anxiety disorders. They are twice as likely as men to experience most anxiety disorders [28]. In our study, the median scores of STAI, s-STAI, and t-STAI were higher among women, which means they have higher levels of anxiety in general, anxiety as a state and anxiety as a trait than men. This was further confirmed by logit models. Mohsen at al. assessed mental health among Syrians during war and the COVID-19 pandemic. Their study used the seven-item Generalized Anxiety Disorder (GAD-7) scale and found that women were more likely to suffer from anxiety disorders than men [29]. Women are believed to be more prone to developing anxiety disorders in emergency circumstances. During the war in Ukraine, anxiety levels among young adults in Central Europe were studied. Researchers found that women had higher mean GAD-7 scores than men [30]. Similar results were obtained using the same scale in a study by Elhadi et al., who assessed anxiety levels among Libyan medical students during the civil war and COVID-19 pandemic. They revealed that women had higher levels of anxiety than men [31]. These findings were also supported by a study by García-González et al. that investigated anxiety among nursing students during the COVID-19 pandemic [32].

Elmer at al. conducted a study on the mental health of students in Switzerland before and during the COVID-19 pandemic. Depressive symptoms, anxiety, stress, and loneliness were assessed. The results obtained showed that in a crisis situation like the COVID-19 pandemic, the mental health status of students was worse than before the pandemic. In addition, this study found that gender was not significantly associated with changes in mental health [33]. In contrast, our study found that there was a significant, very weak correlation between total STAI and gender, s-STAI and gender, and t-STAI and gender.

Ramón-Arbués et al.’s study conducted among Spanish students concerned determining the prevalence of depression, anxiety and stress and their associated indicators using the Depression, Anxiety and Stress Scale (DASS-21). They showed that age under 21 was significantly related to anxiety, depression and stress symptoms [34]. Similar results were obtained in a study conducted in the Philippines, which found that age-group of 12–21.4 years had significantly higher levels of anxiety. Moreover, the researchers showed that students reported more anxiety symptoms than employed people [35]. In our study, we considered the year of study, and if a person was a student of at least the fourth year, then the chance of increased STAI decreased. This applies to the STAI total score and anxiety as a state. A survey of medical students during the Syrian war assessing, among others, anxiety, showed that studying in the fifth or sixth year was associated with a lower likelihood of experiencing anxiety than studying in the second year [36]. Interesting results were obtained by Quek et al. when they reviewed the literature to assess the prevalence of anxiety among medical students. Their work showed that the global prevalence of anxiety was higher among clinical students than among preclinical students. Thus, it seemed reasonable to conclude that the prevalence of anxiety was higher among older students than among younger students [37].

The logit models in our survey showed that if a respondent had savings in the form of money, then the chances of an increase in the STAI survey score decreased. Regarding finances, a study of Libyan medical students found that having a steady financial income was not statistically associated with anxiety symptoms [31]. A study by Ward et al. among German medical students found that financial problems were associated with poor mental health [38]. Similarly, a study of British students found that financial hardship could increase anxiety levels and thus affect academic achievement [39].

Our study showed that the correlations between STAI scores and religion were insignificant and very weak (for STAI total: *p* = 0.479; s-STAI: *p* = 0.3998; t-STAI: *p* = 0.526). In contrast, Gao et al. conducted a study on anxiety among Chinese students. Their study found that religion (Buddhism and Christianity) was significantly associated with anxiety. As for other religions, the result was statistically insignificant (*p* = 0.372) [40]. In the present study it was shown using logit models that if a person professed Christianity, the chance of increased STAI total score, and therefore anxiety level, decreased by over 40%. Interestingly, a study of Mexican students showed that not professing any religion is one of the predictors of higher levels of anxiety [41]. Religion helps reduce stress and also improves coping behavior during a time of crises. This is confirmed by a study that found that religion was the most commonly used coping strategy (assessed using the Brief-Coping Orientation of Problem Experienced (COPE) inventory scale) during a crisis like the COVID-19 pandemic among Saudi nursing students [42].

The factors shown in this study that increase and decrease the odds of anxiety in students should also be presented in a practical context, as they could become ways for intervention and support. Being a senior student decreased the odds of an increase in anxiety, both in the STAI total and in the s-STAI, suggesting that a potential group to be targeted for psychological support could be lower-year students. Being a Christian also reduced the odds of an increase in anxiety (STAI total and t-STAI). This suggests that meetings could be held for students to discuss religious, ethical and moral issues. Such meetings would be held with specialists (such as psychologists, ethicists, theologians) for all willing students, respecting their other religions. Having savings also reduced the odds of increased anxiety (STAI total). Savings can provide a sense of material security and reduce worries about monetary deficiencies, which translates into less anxiety in an anxious situation. Note that if a person feared an armed attack by Russia on Poland, then there was an increased chance of increased anxiety (STAI total, s-STAI, t-STAI). In these cases, the help of psychologists who would work with students with war-related anxiety would be suggested.

There are some limitations to our study. First, we used an online questionnaire, which may have introduced some reporting bias. In addition, we would like to highlight that data collection took place from March 1 to March 30, when the war had been going since 24 February 2022, so based on our study, no conclusions about anxiety during the entire war can be drawn [43]. Moreover, predominance of the male gender and first year students in the study may affect the distribution of the results. This study did not take into account other factors, such as a family history of mental illness, emotional trauma, pressure during studies, or family support.

We believe that this could be used for further research depicting anxiety levels in a larger study group in different populations. In the future, a study could also be conducted on a similar population and evaluate anxiety levels at two time points. We would like our study to show subgroups of students who could potentially benefit from the help of psychologists due to their anxiety levels.

## 5. Conclusions

The hypotheses were positively verified.

Higher levels of anxiety were found in females compared to males. The highest level of anxiety overall was found in year 1 students, the lowest in year 5 students. The highest level of anxiety as a state was found in year 3 students, the lowest in year 6 students. The highest levels of anxiety as a trait were found in year 6 students, the lowest in year 2 students. The highest level of anxiety overall was found in pharmacy students, the lowest in midwifery students. The highest level of anxiety as a state was found in nursing students, the lowest in midwifery students. The highest level of anxiety as a trait was found in pharmacy students, the lowest in midwifery students. A relatively strong relationship was found between anxiety and gender. Protective factors reducing the odds of increasing anxiety included being a senior student, being a Christian, and having savings. In contrast, risk factors that increased the odds of increasing anxiety included being a female and fear of an armed attack by Russia on Poland. This study shows the groups of students who could benefit from the help of specialists. On the other hand, the study could also be useful for specialists such as psychologists, ethicists, and theologians, as it points to immediate groups of students who could benefit from their help to find the right way to cope with war-related anxiety.

## Figures and Tables

**Table 1:** Comparison of the percentage of men and women in terms of answers to the questions.

			Percentage of		
Variable		Participants (*n* = 510)	Female (*n* = 330)	Male (*n* = 180)	*p*-Value
Are you afraid of an armed attack by Russia on Poland?	Yes	52	39	13	- *
	No	48	40	63	0.0003
Are you stocking up?	Yes	25	26	26	- *
	No	74	74	74	- *
Do you have cash savings?	Yes	74	73	75	0.6697
	No	26	17	9	- *
Did you withdraw your savings money?	Yes	19	20	1	- *
	No	81	80	83	0.4508
If you had savings, would you withdraw them from the bank?	Yes	16	11	5	- *
	No	84	83	86	0.4109
In the event of the armed conflict in Poland, would you take an active part in it?	Yes	30	24	42	- *
	No	70	76	58	0.0006
Would you leave Poland in the event of armed conflict?	Yes	61	67	5	0.0046
	No	39	33	50	0.0140
Are you taking an active part in helping Ukraine?	Yes	59	62	53	0.1358
	No	41	38	47	0.0085
In your opinion, is the aid to Ukraine sufficient?	Yes	42	44	3	- *
	No	49	46	53	0.2782

*—values do not meet the test for two structure indicators when comparing the percentage of the number of women and men.

**Table 2:** Logit model parameter evaluations.

Variable	Parameter’s Estimation	*W*	*p*-Value	OR
Intercept	−0.4121	2.0797	0.1493	**-**
Gender	0.9325	18.1889	0.0000	2.5407
Year of study	−0.7874	10.0864	0.0015	0.4550
Are you afraid of an armed attack by Russia on Poland?	1.3335	40.1361	0.0000	3.7944
Religion	−0.5280	6.1812	0.0129	0.5800
Do you have cash savings?	−0.6601	7.7477	0.0054	0.5168

*W*—Wald test, OR—odds ratio.

**Table 3:** Classification accuracy of the logit model.

Qualification of Persons Based on the Model	Actual Affiliation of Persons		Overall Accuracy of the Classification
	$y_{i}=1$	$y_{i}=0$	
${\hat{y}}_{i}=1$	145	68	69.04%
${\hat{y}}_{i}=0$	83	185	
Sensitivity, specificity	63.60%	74.00%	

**Table 4:** Logit model parameter evaluations.

Variable	Parameter’s Estimation	*W*	*p*-Value	OR
Intercept	−1.0790	28.8953	0.0000	-
Gender	0.7509	12.4052	0.0004	2.1189
Year of study	−1.2385	23.8656	0.0000	0.2898
Are you afraid of an armed attack by Russia on Poland?	1.4375	50.3800	0.0000	4.2102

**Table 5:** Classification accuracy of the logit model.

Qualification of Persons Based on the Model	Actual Affiliation of Persons		Overall Accuracy of the Classification
	$y_{i}=1$	$y_{i}=0$	
${\hat{y}}_{i}=1$	158	57	72.27%
${\hat{y}}_{i}=0$	86	207	
Sensitivity, specificity	65.56%	78.41%	

**Table 6:** Logit model parameter evaluations.

Variable	Parameter’s Estimation	*W*	*p*-Value	OR
Intercept	−0.6874	9.5296	0.0020	-
Gender	0.6855	10.5171	0.0012	1.9848
Are you afraid of an armed attack by Russia on Poland?	0.9723	23.3041	0.0000	2.6440
Religion	−0.4004	3.8652	0.0493	0.6701
If you had savings, would you withdraw them from the bank?	0.7659	7.4307	0.0064	2.1509
In the event of the armed conflict in Poland, would you take an active part in it?	−0.4719	4.6814	0.0305	0.6238

**Table 7:** Classification accuracy of the logit model.

Qualification of Persons Based on the Model	Actual Affiliation of Persons		Overall Accuracy of the Classification
	$y_{i}=1$	$y_{i}=0$	
${\hat{y}}_{i}=1$	157	72	67.70%
${\hat{y}}_{i}=0$	84	170	
Sensitivity, specificity	65.15%	70.93%	

## Data Availability

All data were collected in the Department of History of Medicine and Medical Ethics, Pomeranian Medical University in Szczecin.
